# Proteomic Analysis of Human Endometrial Tissues Reveals the Roles of PI3K/AKT/mTOR Pathway and Tumor Angiogenesis Molecules in the Pathogenesis of Endometrial Cancer

**DOI:** 10.1155/2020/5273969

**Published:** 2020-08-22

**Authors:** Zhen Liu, Zhipan Hong, Pengpeng Qu

**Affiliations:** ^1^Department of Obstetrics & Gynecology, Tianjin Medical University, Tianjin 300070, China; ^2^Department of Gynecology, Chifeng Municipal Hospital, Chifeng Clinical Medical School of Inner Mongolia Medical University, Chifeng 024000, China; ^3^Department of Gynecology Oncology, Tianjin Central Hospital of Gynecology & Obstetrics, Tianjin 300070, China; ^4^Department of Tumor Surgery, Chifeng Municipal Hospital, Chifeng Clinical Medical School of Inner Mongolia Medical University, Chifeng 024000, China

## Abstract

As one major gynecological malignancy, endometrial cancer (EC) has been widely studied recently. However, its pathogenesis is still unclear to date. In this study, we identified differentially expressed proteins between 30 endometrial cancer tissues and 30 matched normal controls using 2D LC-MS/MS quantitative proteomics. As a result, we identified 619 differentially expressed proteins among all 2521 proteins being quantified. Further analyses suggested that the changes of fat, amino acid metabolism, peroxisome, extracellular signal, cytoskeleton, and other signaling or metabolic pathways may be closely related to the development of this cancer. Particularly, the PI3K/AKT/mTOR pathway-related molecules including PI3K and mTOR, ERK (the molecule of the ERK pathway), SPP1, and ANGPT2 (angiogenesis-related molecules) are highly associated with the pathogenesis of EC, which were reconfirmed by western blot and immunohistochemistry (IHC) analysis. In summary, our study revealed that the PI3K/AKT/mTOR pathway and tumor angiogenesis molecules contribute to the pathogenesis of endometrial cancer.

## 1. Introduction

Endometrial cancer (EC) is a malignant tumor of uterine epithelial cells. EC generally occurs in postmenopausal women over the age of 50, and the incidence of which peaks at the age range of 50–59. The incidence of EC occupies the first place in gynecological tumors in Europe and America [1]. EC is a multifactor process in which the deletion of tumor suppressor genes (TSGs) and the activation of oncogenes are particularly important [2, 3]. Many epidemiological investigations present that high BMI index, hypertension, metabolic syndrome, diabetes, and long-term hormone use were major potential causes of endometrial cancer. Among them, high progesterone or no progesterone resistance is the primary risk factor [4].

Nowadays, many studies have shown that excessive cell proliferation, inactivation of TGSs, activation of oncogenes, and abnormal signal conducts might be critical in various stages of EC such as onset and development. For example, excessive secretion of TGF-beta 1 (TGF-*β*1) can promote the process of malignant cell transformation by stimulating the extracellular matrix formation, increasing the tumor angiogenesis, and inducing excessive cell growth [5]. EC is known to be a kind of estrogen-dependent tumors. Estrogen increases the activity of Wnt signal by promoting the expression of Wnt and followed by increasing the expression of its downstream molecular C-myc, which may cause EC [6, 7]. Meanwhile, the mutation of molecules in Wnt/*β*-catenin signaling, including Wnt, *β*-catenin, APC, and Axin, also induces the malignant transformation process of endometrial tissue [8]. Otherwise, abnormal cell cycle function might be a role in the occurrence of EC disease. Studies have confirmed that overexpression of Cyclin D1 was found in 51. 4% EC patients, which will accelerate cell cycle and promote cell proliferation by increasing the formation of Cyclin D1 and CDK4/6 complex [9]. Although nowadays great progress has been made in the research on the pathogenesis of EC disease, the underlying mechanism is still not fully clear.

Proteomics, which was first introduced by Marc Wilkins in 1996 to denote the “PROTein complement of a genOME,” represents the characterization of proteome including protein expression, structure, functions, interactions, and modifications [10]. Proteomics can also reflect the real-time changes of protein in the cell, which provides us with high-throughput and high-sensitive technical strategies in the etiology of disease, necessary for disease early diagnosis, prognosis, and monitoring [11–14]. In fact, with the support of high-throughput technologies, proteomics has become one of the most significant methodologies to understand the processes of complex biochemical and explore the gene function [15–17].

Proteomic studies have been performed in several kinds of cancers. For example, Pan et al. provided the detailed information of proteome alterations in bodily fluids of pancreatic cancer patients [18]. Bohnenberger et al. studied the diagnostic proteomics of pulmonary head-and-neck cancer (HNSCC), providing the proteomic resource for HNSCC and squamous cell lung cancer (SQCLC) [19]. Chauvin and Boisvert described a method to compare protein expression and subcellular localization in different stages of colorectal cancer, which enables an integrated analysis of different kinds of cell lines and the function of pathways that cancer cell lines were involved in [20].

In the present study, we provided an effective tool to seek for the biomarkers with excellent specificity and sensitivity for EC disease based on proteomics. Here, by applying isobaric tag for relative and absolute quantitation (iTRAQ) coupled with 2D-LC MS/MS, we confirm that activation of PI3K/AKT/mTOR and excessive angiogenesis might be involved in the occurrence and development of EC. The results suggest that inhibiting the PI3K/AKT/mTOR and angiogenesis might be an effective therapeutic strategy in the treatment of EC disease.

## 2. Materials and Methods

### 2.1. Reagents

Isobaric tags for relative and absolute quantitation (iTRAQ) Reagent Multiplex Kit (4352135) were purchased from AB Sciex (Foster City, CA). Biotin-Streptavidin HRP Detection Systems and Diaminobenzidine (DAB) Kit (ZLI-9018, China) were from ZSGBBio Company (Beijing, China). Acetonitrile (14261) and formic acid (56302) were from Sigma-Aldrich. Anti-ANGPT2 (ab155106), anti-SPP1 (ab214050), anti-ERK (ab17942), anti-pERK (ab214362), anti-mTOR (ab2732), anti-pmTOR (ab109268), and anti-PI3K (ab40776) were from Abcam.

### 2.2. Ethics Statements

This study was approved by the Medical Ethics and Human Clinical Trial Committee of Chifeng Municipal Hospital. All experiments were performed in accordance with the Helsinki Declaration. Prior to recruitment, we acquire from each participant a written informed consent. The materials used in this study were collected at the department of gynecology of Chifeng Municipal Hospital (Inner Mongolia, China) in 2018-2019.

### 2.3. Clinical Samples

The endometrial tissues provided by Chifeng Municipal Hospital (Inner Mongolia, China) and 30 health maternal and 30 EC cases were provided by mentioned hospitals.

### 2.4. Quantitative Proteomics

**Figure 1:**
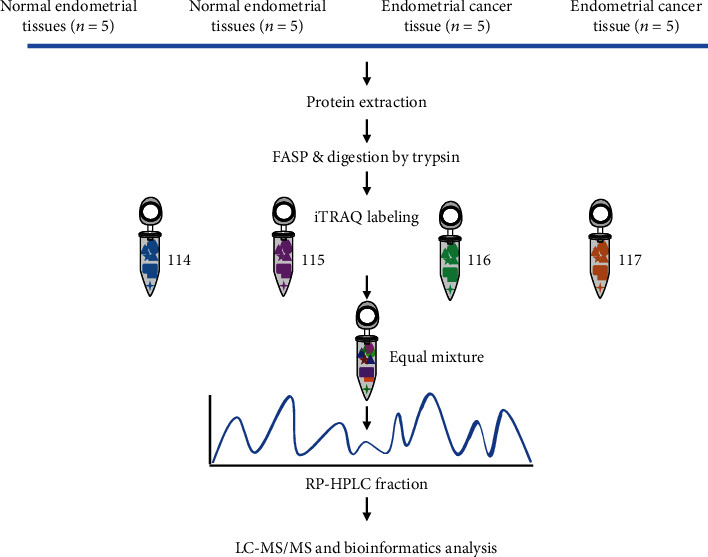
Schematic workflow of materials and methods. Samples from endometrial tissues of NE (*n* = 10) and EC cases (*n* = 10) were collected in two biological replicates. Similar amounts of proteins were digested into peptides using Trypsin and Lys-C Mix enzymes. The digested peptides were subsequently extracted and desalted. All samples were pooled together after 4-plex iTRAQ labeling and analyzed using 2D LC-MS/MS. Tag 116 and 117 for endometrial tissues of normal cases and 114 and 115 for endometrial tissues of EC cases, respectively.

The potential changes between EC and health maternal groups were analyzed using iTRAQ combined with the two-dimensional liquid chromatography-tandem mass spectrometry (2D LC-MS/MS) (Figure 1) [21]. To ensure the accuracy of quantitative proteomics, we divided the tissue samples into 4 groups, including 2 normal groups and 2 EC groups. To reduce the individual differences and homogenates, 5 independent endometrial tissues in each group were pooled together and then were lysed with the lysis buffer (8 M urea, 5 mM IAA, 50 mM NH_4_HCO_3_, and protein inhibitor cocktail), which was followed by sonication and centrifugation. We used the BCA assay to determine the appropriate protein concentration. The equal amount of lysates from each of the pooled tissue sample (~100 *μ*g) was reduced with dithiothreitol (DTT) and alkylated with iodoacetamide (IAA). The method of Trypsin/Lys-C Mix digestion in the gel was performed according to the previous description [22]. Briefly, 100 *μ*g peptide samples from normal-1 and 2 and EC-1 and 2 were labeled by iTRAQ4-114, 115, 116, and 117. Differentially labeled peptides were equally mixed, dried with a speed-vac, and then desalted with Sep-Pak C18 Vac cartridges. The labeled samples were separated into 20 fractions with an offline Agilent 1100 HPLC System with Xbridge®Peptide BEH C18 column (3.5 *μ*m, 4.6 × 250 mm, Waters, Milford, MA). The fractionated peptide was measured by using LC-MS/MS on an Orbitrap Fusion mass spectrometer (Thermo Fisher Scientific, Waltham, MA, USA), which was equipped with an EASY-nLC1000 ultra-high-pressure liquid chromatography (HPLC) system (Thermo Fisher Scientific, Waltham, MA, USA). The peptides were dissolved in buffer A with 0. 1% formic acid and 2% acetonitrile in water and loaded on a homemade C18 capillary column (75 *μ*m ID × 15 cm). After that, the peptides were eluted with a linear 60 min gradient in an 8-38% buffer B with 0.1% formic acid in 90% acetonitrile, which was followed by 38%-80% buffer B for 6 min and 80% buffer B for 4 min at a flow rate of 300 nL/min. We then ionized the eluted peptides using a nanospray ion source (NSI) with 2.2 kV voltage. For the full MS scan, peptides were measured by the Orbitrap analyzer with the mass range setting to be 450-1500 *m*/*z*, and the resolution being 60,000 at *m*/*z* 200. We set the automatic gain control (AGC) target to 5 × 105, and the maximum injection time was set to 50 ms. We fragmented the top 15 most intense ions via high-energy collision-induced dissociation (HCD) with 40% normalized collision energy (NCE). The fixed first mass of 100 *m*/*z* was used for MS/MS scans in the Orbitrap analyzer (resolution was 15,000 at *m*/*z* 200. The AGC target was set to 5 × 105, the isolation width was 1 *m*/*z*, the dynamic exclusion range was the 60 s, and the maximum ion injection time was set as 80 ms. The raw data were analyzed using the MaxQuant software suite (version 1.5.1.2). The search engine was against the UniProt human reference protein database (version June 2016). And the results of bioinformatics analysis were visualized using R (version 3.2.3) package GOplot1.1 [23]. Cytoscape 3.3.0 software was used to assign the interaction network of the altering expression proteins [24].

### 2.5. Western Blot and Immunohistochemistry (IHC) Analysis

The western blot and IHC analysis were performed according to the previous description [25]. Briefly, 60 *μ*g of protein samples was electrophoresed by 10% SDS-PAGE and transferred onto 0.22 *μ*m nitrocellulose membrane by a semidry electroblotter. After incubation, the protein bands were visualized using the ECL, reagent, and quantified with the Scion Image [26]. IHC staining was performed with Biotin-Streptavidin HRP Detection Systems using placenta tissue slices, which contained 30 cases of EC and 30 cases of health endometrial tissues. In brief, endometrial tissues were cut into 5 *μ*m slices, and after deparaffinized, hydrated, the slices were incubated with 3% H_2_O_2_ to block endogenous peroxidase. We used heat-induced epitope retrieval methods to retrieve the antigen. At room temperature, we permeabilisate the slices with 0. 25% Triton X-100 for 10 min and blocked with 1% BSA for 1 h. After that, we incubated the slices with primary antibody (1 : 100) overnight and then with secondary and anti-rabbit IgG peroxidase for 15 min followed by incubation with DAB substrate solution. The slices were stained with hematoxylin and dehydrated. 10 digital images at 200x magnification were captured using the Olympus CX-31 microscope (Olympus). The protein expression levels were assessed by the mean optical density (MOD) value, which was defined to be the ratio between the integrated integral optical density (IOD) and the sum of all areas.

### 2.6. Statistical Analysis

The standard deviation (SD) was calculated by fitting the Gaussian distribution for the quantitative proteomics data. Other data are described as the mean ± S.E.M. The differential proteins between cancer and normal groups were achieved by Student's test; the *p* values were adjusted by the Benjamini-Hochberg method; a protein was considered differential if (1) its adjusted *p* value is less than or equal to 0.05 and (2) |log_2_^ratio^| is greater than or equal to 1.

## 3. Results

### 3.1. There Are Thousands of Proteins Quantified and Hundreds of Differential Proteins between EC and Controls

**Figure 2:**
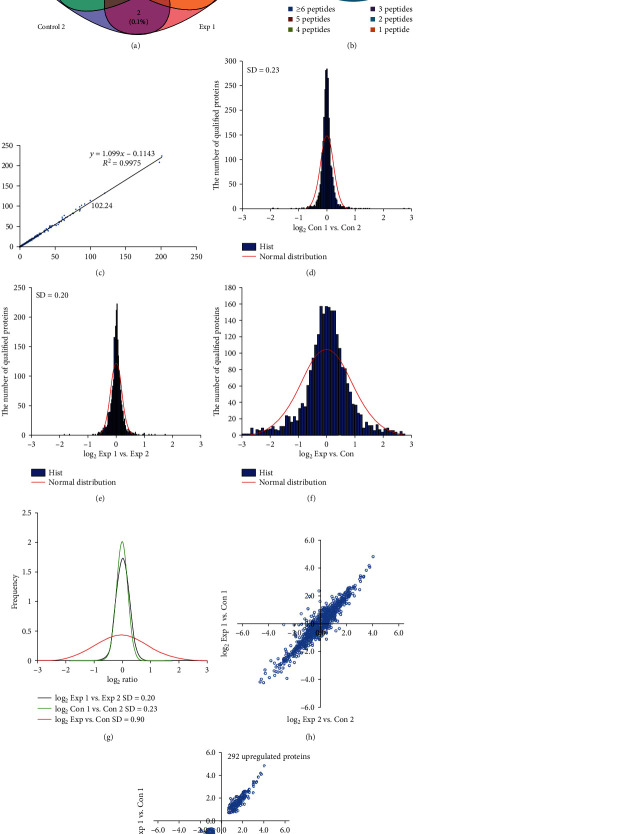
The distribution of quantitative proteomics using Gaussian fit and determination of altering proteins. (a) The Venn diagram representing the overlap of quantified proteins in two biological replicates, respectively. (b) Histogram showing the number of peptides matched to proteins. The *x*-axis displays the number of identified peptides. The primary *y*-axis indicates the number of identified proteins (bars). The second *y*-axis represents the percent (lines). (c) Scatter plots of the two biological replicates of Con 1 and Con 2, iTRAQ 116, 117-labeled Exp 1 and Exp 2. (d) Histograms of log_2_ ratios of the abundance of qualified proteins between Con 1 and Con 2 (*n* = 15). (e) Histograms of log_2_ ratios of the abundance of qualified proteins between Exp 1 and Exp 2 (*N* = 15). (f) Histograms of log_2_ ratios of the abundance of qualified proteins between Exp and Con (*n* = 15). (g) The comparison of log_2_ ratio distributions of quantified proteins in (a) to (c). (h) Global distribution of the overlap of the qualified proteins in twice independent repeat experiment (*n* = 15). (i) Distribution of 619 differentially expressed proteins in twice independent repeat experiments. Of these, 327 proteins exhibited downregulated expression and 292 proteins exhibited upregulated expression.

MS-based quantitative proteomics strategy was appropriate for comparing the difference in endometrial tissues (Figure 1). Owing to the interference with abundant protein in high abundance serum disrupt of endometrial tissues, we only identified 2600 proteins, among which 2521 were successfully quantified with false discovery rates (FDRs) of both peptides and proteins lower than 1% (Figure 2(a)). Among those proteins identified, 34.1%, 21.7%, 11.6%, 8.2%, 5.1%, and 19. 4% of proteins comprised of 1 peptide, 2 peptides, 3 peptides, 4 peptides, 5 peptides, and at least 6 peptides, respectively (Figure 2(b)). The correlation index R2 of MS intensity between 114- and 115-labeling peptides was 0.9975 (Figure 2(c)). The distribution of the log_2_^Con 1/Con 2^ ratio could be fit to a Gaussian curve with an SD of 0.23 (Figure 2(d)), and the distribution of log_2_^Exp 1/Exp 2^ ratios could be fit to 0.20 (Figure 2(e)). Meanwhile, the SD value was 0.90 in Exp versus Con by the same mean (Figure 2(f)). The SD value of Exp versus Con was significantly increased compared to those of two groups (*p* < 0.05) (Figure 2(g)). The two quantitative results are presented in two plots log_2_^Exp 1/Con 1^ ratios of the proteins versus the log_2_^Exp 2/Con 2^ ratios and the changing expressed proteins that showed a similar trend and presented in the first or third quadrant (Figure 2(h)). We defined a protein to be upregulated if its expression in the EC group is greater than that in the control group and downregulated otherwise. As a result, we identified 619 differentially expressed proteins, including 327 downregulation expression proteins and 292 upregulation expression proteins (Figure 2(i)).

### 3.2. Gene Ontology Analysis and Network Analysis Reveal That the Pi3k-Akt Signaling Pathway Might Play Critical Roles in EC

**Figure 3:**
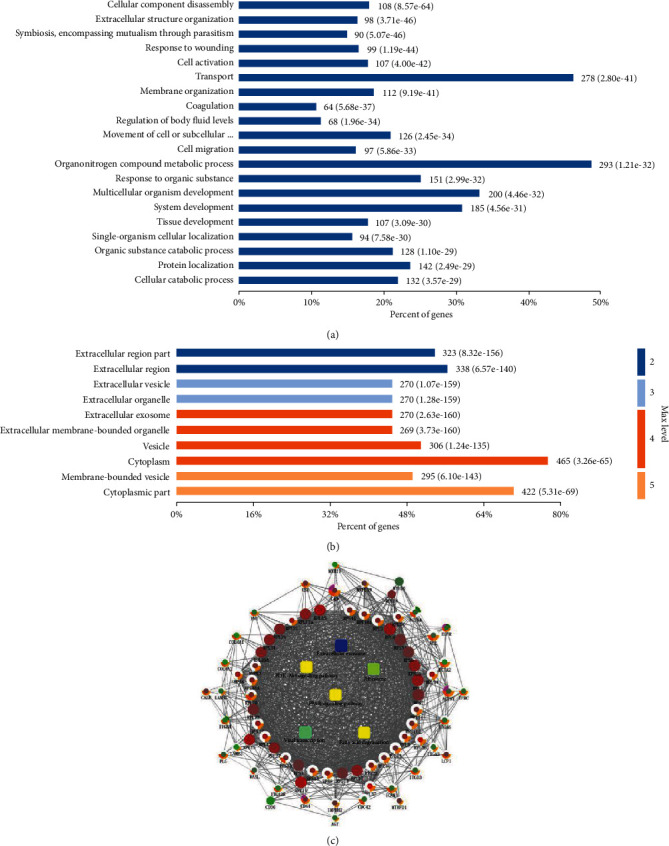
Bioinformatics analysis for differentially expressed proteins in endometrial tissues between EC and normal cases. (a, b) Diagram showing the biological processes (BP) and cellular components (CC) of altering proteins using DAVID (version 6.8) analysis and R (version 3.2.3) package GOplot (version 1.1). (c) Network of protein-protein interaction (PPI) information was produced using the CluPedia plugin of the Cytoscape (version 3.3.0).

By the enrichment analysis with Gene Ontology (GO) terms, difference expressed proteins were involved in many biological processes (BP), such as in extracellular matrix, injury repair, cell activation, coagulation, and phylogeny (Figure 3(a)); meanwhile, these molecules were also enriched in many cellular components (CC), such as extracellular matrix, vesicle transport, and cell membrane structure (Figure 3(b)).

The network analysis of changing proteins was mapped using Cytoscape 3.3.0 software (Figure 3(c)). These proteins were mainly involved in extracellular exosome, Pi3k-Akt signaling pathway, ribosome, PPAR signaling pathway, viral transcription, and fatty acid degradation; the PI3K-Akt signaling pathway plays an important role in the development of EC and is related to the angiogenesis. At the same time, we found two molecular proteins with a strong correlation with angiogenesis: SPP1 and ANGPT2.

### 3.3. The Proteomic Alteration of mTOR and Other Proteins Was Validated by Western Blot and IHC

**Figure 4:**
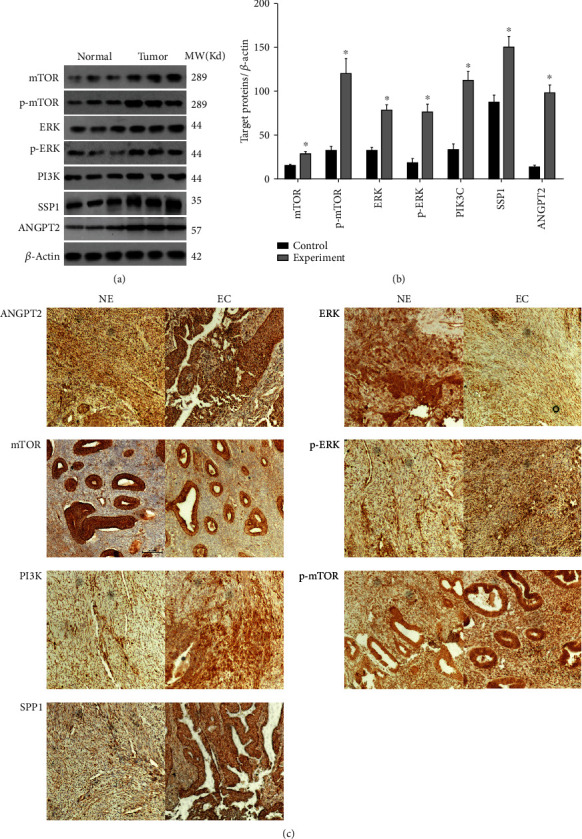
Validation of biological processes in EC and NE tissues. (a, b) Western blotting analysis showed that the expression level of ANGPT2, ERK, mTOR, p-ERK, PI3K, p-mTOR, and SSP1 of endometrial tissues in EC patients was significantly increased. (c) The IHC results further confirmed these altering trends. Data were described as mean ± S.E.M.

Figures 4(a) and 4(b) confirmed a significant increase in mTOR, p-mTOR, ERK, p-ERK, PIKEC, SSP1, and ANGPT2 in the EC patients compared with those of the NE group (*p* < 0.05). Meanwhile, the IHC results also confirmed these altering trends (Figure 4).

## 4. Discussion

In the present study, we successfully screened 619 altering proteins between EC and NE tissue samples; these molecules were enriched into many important various biological processes, such as amino acid metabolism, peroxidase body, extracellular signals, cytoskeletal signals, and metabolic pathways. Meanwhile, the altering expressed proteins also were involved in many vital signaling pathways, including PI3K-AKT-mTOR, PPAR, and AGE-RAGE.

We noticed that there was a close relation between the occurrences of EC and the activation of the PI3K-AKT-mTOR signaling. The PI3K-AKT-mTOR pathway plays a critical role in the regulation of the physiological process in normal cells [27]; it also was a key signal for protooncogene activation to promote tumor cell proliferation and metastasis. PI3K was a dimer protein composed of subunit Pll0 and regulatory subunit P85, which had a dual activity of lipid kinase and protein kinase [28]. Activating ERK signaling would increase the bind ability between Pll0 and Ras and then activated the PI3K followed by increased PIP3 product, which was associated with an intracellular signaling protein AKT with PH2 domain and increased its phosphorylation level. p-AKT could phosphorylate the GLUT4 and GSK3*β* to promote glucose metabolism and regulates cell cycle [29–33]. Moreover, AKT also could phosphorylate the tuberous sclerosis complex (TSC1/2) to increase the release of the RHEB followed by activating the mammalian target of rapamycin (mTOR). mTOR belonged to an important cell signal; its stability affects the expression of cytokines, transcription, and protein synthesis and regulates cell growth, autophagy, and apoptosis. mTOR had been identified as a new target for cancer therapy, which was found to be activated in many cellular processes, including insulin resistance, adipogenesis, tumor formation, angiogenesis, and lymphocyte activation. The mTOR inhibitors (rapamycin and its analogs) had been used in the treatment of diseases such as solid tumors, organ transplantation, and rheumatoid arthritis [34, 35]. In this study, we found that the expression levels of PI3K, AKT, and mTOR in EC tissue samples were significantly increased compared with those in control tissue samples. This evidence suggested that there was a close relation between the occurrence of EC and activating the PI3K-AKT-mTOR signaling.

Many studies showed that activating mTOR signaling would promote a variety of pro-angiogenesis-related protein expressions, including HIF1*α*, VEGF, PDGF, FGF, and TGF*β* in various types of tumor tissues. In the present study, the expression levels of SPP1 and ANGPT2 in EC tissue samples were significantly increased compared with those in control samples. Studies had shown that SPP1 is an important role in VEGF-promoting angiogenesis by enhancing the migration ability of vascular endothelial cells [36, 37]. ANGPT2, as a member of the secretion cytokine family, was active in several important biological processes, including vascular remodeling, wound repair, and tumor angiogenesis [38, 39]. The overexpression of SPP1 and ANGPT2 in EC tissue samples also indirectly confirmed that abnormal tumor angiogenesis was closely related to the occurrence of EC by activating the tumor angiogenesis. Otherwise, we also noticed that the expression of oncogene ERK and p-ERK in EC samples was significantly increased compared with those in control samples. ERK is a member of the mitogen-activated protein kinase (MAPK) family. It plays critical roles in the MAPK signal pathway, which involves many cellular processes, such as proliferation, transcription regulation, and differentiation through the regulation of transcription, translation, and translocation [40, 41].

There are a few limitations of this study. For example, the identified EC-associated genes and pathways were only validated by western blot and immunohistochemistry (IHC) analysis. A more convincing experimental validation at a cell line and mouse model is needed. In addition, we only examined the single protein expression differences between the EC group and the normal group. A more detailed analysis using protein coexpression and expression network might be useful in finding gene regulations altered by EC.

## 5. Conclusion

Based on quantitative proteomics, we found that activating the PI3K-AKT-mTOR and excessive tumor angiogenesis might contribute to the occurrence of EC disease. However, more in-depth research needs to be further performed in the etiology of EC disease in in vivo and in vitro experiments.

## Data Availability

The datasets generated/analyzed during the current study are available.

## Abbreviations

EC: Endometrial cancer
TSGs: Tumor suppressor genes
TGF-*β*1: TGF-beta 1
FDR: False discovery rate
GO: Gene ontology
BP: Biological processes
CC: Cellular components
MAPK: Mitogen-activated protein kinase.
